# African Vultures Don’t Follow Migratory Herds: Scavenger Habitat Use Is Not Mediated by Prey Abundance

**DOI:** 10.1371/journal.pone.0083470

**Published:** 2014-01-08

**Authors:** Corinne J. Kendall, Munir Z. Virani, J. Grant C. Hopcraft, Keith L. Bildstein, Daniel I. Rubenstein

**Affiliations:** 1 Department of Ecology and Evolutionary Biology, Princeton University, Princeton, New Jersey, United States of America; 2 Ornithology Section, Department of Zoology, National Museums of Kenya, Nairobi, Kenya; 3 The Peregrine Fund, Boise, Idaho, United States of America; 4 Frankfurt Zoological Society, Arusha, Tanzania; 5 Acopian Center for Conservation Learning, Hawk Mountain Sanctuary, Orwigsburg, Pennsylvania, United States of America; Bangor University, United Kingdom

## Abstract

The ongoing global decline in vulture populations raises major conservation concerns, but little is known about the factors that mediate scavenger habitat use, in particular the importance of abundance of live prey versus prey mortality. We test this using data from the Serengeti-Mara ecosystem in East Africa. The two hypotheses that prey abundance or prey mortality are the main drivers of vulture habitat use provide alternative predictions. If vultures select areas based only on prey abundance, we expect tracked vultures to remain close to herds of migratory wildebeest regardless of season. However, if vultures select areas where mortality rates are greatest then we expect vultures to select the driest regions, where animals are more likely to die of starvation, and to be attracted to migratory wildebeest only during the dry season when wildebeest mortality is greatest. We used data from GSM-GPS transmitters to assess the relationship between three vulture species and migratory wildebeest in the Mara-Serengeti ecosystem. Results indicate that vultures preferentially cluster around migratory herds only during the dry season, when herds experience their highest mortality. Additionally during the wet season, Ruppell’s and Lappet-faced vultures select relatively dry areas, based on Normalized Difference Vegetation Index, whereas White-backed vultures preferred wetter areas during the wet season. Differences in habitat use among species may mediate coexistence in this scavenger guild. In general, our results suggest that prey abundance is not the primary driver of avian scavenger habitat use. The apparent reliance of vultures on non-migratory ungulates during the wet season has important conservation implications for vultures in light of on-going declines in non-migratory ungulate species and use of poisons in unprotected areas.

## Introduction

The study of animal ecology has focused on herbivores, predators and parasites, and has largely overlooked scavengers [1]. Unlike herbivores, whose ecology is often governed by the interplay of predation risk and forage availability [2], [3], [4], or predators whose habitat use may be determined more by prey accessibility than abundance [5], [6], scavengers face a different set of challenges and their ecology is likely to be mediated by other ecological factors. In many ways scavengers provide an extreme example of a meta-community – one that assembles, competes, and disassembles over short periods of time as a carcass is broken down [7]. Scavengers must overcome the spatial and temporal challenges of feeding on carrion, an ephemeral and generally patchily distributed resource often used by large numbers of potential competitors, including predatory facultative scavengers [1], [8]. Foraging success for scavengers depends on their ability to search across large areas and rapidly detect carrion before it is decomposed by microbes or consumed by invertebrate and vertebrate competitors [9]. Because they generally experience limited predation, food availability, its predictability, and its accessibility have generally been found to be the key factors determining scavenger habitat use and distribution [10], [11], [12]. For scavengers relying solely on carrion, food availability is a factor of not just live-prey abundance but also prey mortality, which vary both spatially and temporally. The relative importance of prey abundance and mortality and the interactions between these two factors, although likely to shape habitat use in scavengers, have not been explored.

Vultures (Accipiridae and Cathartidae) are the only obligate vertebrate scavengers [13]. These scavengers feed primarily on carrion from non-predation mortalities, such as those resulting from starvation and disease [14]. Because they soar, vultures can maintain extremely large foraging ranges, even when breeding, and thus effectively track herds of migratory ungulates year-round [15], [13], [8]. Herds of migratory Western white-bearded wildebeest (*Connochaetes taurinus*) represent the highest abundance of live ungulate prey in the Mara-Serengeti ecosystem of East Africa. The region has a distinct and steep rainfall gradient with considerable asynchrony in rainfall patterns across the area [16]. Migratory ungulates maintain superabundance by following rainfall gradients to maximize intake of seasonally available grasses and minimize exposure to predation [17], [18], [19]. In wildebeest, adult mortality peaks during the dry season [20], whereas high neonatal mortality, which accounts for the largest numeric loss in the species is not related to forage availability during the dry season [20]. The approximately twenty-five percent of the 250,000 wildebeest calves born each year in the region die within twelve months and thus represent a substantial food resource for scavengers.

Historic studies on vultures involving marked individuals and radio telemetry suggested that vultures followed herds of migratory ungulates. However results were limited by small sample sizes, limited re-sightings, and the short duration of telemetry devices used [21], [22]. Studies of vultures in Mara-Serengeti indicate that abundance is highest in areas near migrating ungulates and have concluded that the birds follow the migratory herds [15]. Selva and Fortuna [8] suggest that obligate avian scavengers are particularly well-adapted to using aggregated food sources, created by large pulses in carcass abundance, such as starvation of migratory ungulates during the dry season.

Three species of vultures make up the bulk of the avian scavenger guild in the region. Given their dependence on a common resource, the mechanisms that enable coexistence of Lappet-faced (*Torgos tracheliotos*), White-backed (*Gyps africanus*) and Ruppell’s vultures (*Gyps rueppellii*) are not well understood [23]. Coexistence of the two *Gpys* species, in particular, is difficult to explain given the similarity in their feeding and social behavior [24], [25], [26], [23]. The species do differ in breeding behavior with White-backed and Lappet-faced vultures frequently nesting in trees from April to July in the Mara-Serengeti area, whereas Ruppell’s vultures have seasonal variation in breeding season and are cliff-nesting, and suitable cliffs are not common in the ecosystem [22], [27], [28], [29], [30].

Here we determine the main drivers of large-scale habitat use in East African vultures based on data collected from GSM-GPS transmitters attached to Lappet-faced, White-backed, and Ruppell's vultures in the Mara-Serengeti ecosystem of East Africa. In particular, we use our tracked vultures to assess how the spatial and temporal distributions of prey abundance and Normalized Difference Vegetation Index, a proxy for prey mortality, affect vulture habitat use. Based on the theory of an ideal free distribution, we predict that scavengers will select foraging areas where they are the most likely to find carrion [31]. The hypotheses of prey abundance versus prey mortality as the main drivers of vulture habitat use provide alternative predictions: if vultures select areas based only on prey abundance, we expect tracked vultures to remain close to the migratory wildebeest regardless of season. However, if vultures select areas where mortality is greatest then we expect vultures to use the driest regions, where animals are more likely to die of starvation, and to be attracted to migratory wildebeest only during the dry season when mortality is greatest [20].

African vultures are declining rapidly and decreases in Masai Mara National Reserve have been substantial [32], [33]. A more complete understanding of what drives scavenger habitat use will significantly expand existing knowledge about scavenger ecology, help explain coexistence of these similar vulture species, and aid in their protection.

## Methods

### Ethics

Research was conducted in Masai Mara National Reserve (01°05’ S, 34°50’ E), Kenya and was covered under research permit number NCST/5/002/R/448 issued by the National Council for Science and Technology in Kenya. We are indebted to the Narok County Council and the staff of the Masai Mara National Reserve, in particular the wardens Mr. Sindiyo and Mr. Minis for their assistance and permission to conduct vulture research in the reserve. Vultures were trapped using nooses, set up as grids or in a line, along and on top of carcasses [34]. Noose lines and girds consisted of 10 to 20 nooses. Noose grids were made of 90-kg-strength monofilament fishing line. Noose on noose lines were made of coated wire cord or monofilament, and the noose line was made of parachute cord. Nooses were 10–15 cm in diameter. Noose grids were generally staked into the ground using tent stakes, whereas noose on noose lines were tied to carcasses and staked into the ground using 5-cm nails for added stability. Grass or carrion was used to help hold the nooses upright to increase the chance of a capture.

Processing captured birds took approximately 30 minutes; the birds’ eyes were covered to reduce stress and a handler restrained both feet and head. The majority of birds captured in this study were adults, but several sub-adults and one fledgling Lappet-faced Vulture also were tagged and tracked. Age was determined based on plumage and coloration following Mundy et al. (1992). Units were attached as backpacks using 11-mm Teflon ribbon (Bally Ribbon Mills, Bally, Pennsylvania, U.S.A.) following procedures similar to other vulture studies [35], [36] and weighed between 100 and 160 g, or about 2% to 3% of the body mass of the vulture. Backpacks used to attach transmitters were designed to fall off within several years, as recapture of tagged individuals is not likely. Whenever possible, wing tags were attached to aid with the visual identification of individuals in the field. Individually numbered plastic wing tags were attached to the patagium of one wing using cattle ear tags following Wallace et al. [37]. All work with animals was conducted following appropriate protocols and was approved by IACUC at Princeton University under protocol number 1751.

### Study area

East Africa has high wildlife densities and few human-mediated sources of carrion, making it an ideal study system in which to investigate natural scavenger behaviors. The Mara-Serengeti ecosystem is unique because it maintains one of the few ungulate migrations remaining in the world [38], [39].

The Mara-Serengeti ecosystem has the largest ungulate migration in the world, with 1.3 million Western white-bearded wildebeest, 180,000 Burchell’s zebra (*Equus burchelli*), and 250,000 Thomson’s and Grant’s gazelle (*Eudorcas thomsonii* and *Nanger granti*) moving between Serengeti National Park, Tanzania, and Masai Mara National Reserve, Kenya, each year. As a result, this ecosystem is arguably one of the most important areas for scavengers in Africa, supporting high densities of vultures of many species [40]. Rainfall is generally seasonal, with the long rains falling from early February to the end of April, and short rains from November to December [41]. Across the region there is a steep rainfall gradient that increases from southeast to northwest (approximately 400mm to 1200 mm of rain/year) [19].

### Unit deployment

Forty-one battery-powered GSM-GPS transmitters (16 from Africa Wildlife Tracking, Pretoria, South Africa, and 25 from Savannah Tracking Ltd., Nairobi, Kenya) were deployed. Fourteen transmitters were deployed from May to August 2009, 21 from April to October 2010, and 3 in March 2011. These deployments include three re-deployments that occurred after units were recovered from dead birds [42]. Units from African Wildlife Tracking (primarily deployed in 2009) were programmed to record locations four times per day (0300, 1100, 1300, 1500 hours); units from Savannah Tracking Ltd recorded six locations a day (every two hours from 0700 to 1700 hours). Units lasted an average of 8 months (± 0.6 SE).

### Spatial analysis

Analyses were focused on mid-day locations (1100, 1300, 1500 hours) when vultures are most likely to be foraging. Nest sites were established for each individual based on consistent use of an area within a 50 m radius across several months with at least 50 locations in the area. For nests within Masai Mara National Reserve, nest sites were confirmed by spotting the tagged bird on the nest. Days when birds were on the nest during a mid-day point were removed from analysis. To increase independence, a single point was used for each day, which was calculated as the centroid of three mid-day points using ArcGIS 9.3 (Environmental Systems Research Institute, Redlands, California, USA). Data for which these three points were not available in a given day were excluded.

To assess the relationship with ungulate abundance, we related vulture movement to migratory ungulate movement based on 75% kernel polygons representing the distribution of migratory wildebeest from movement data collected over a five-year study for four separate seasons (Wet – January to April, Wet to dry – May to June, Dry – July to October, Dry to wet – November to December) [43]. Proximity between the centroid of a vultures’ daytime range and the wildebeest polygons were calculated and overlapping points were given a value of zero and the nearest distance between boundary of wildebeest polygons and centroid was determined. In addition, a random set of points was also generated for each individual bird within the minimum convex polygon of its overall range (calculated using Hawth’s tools in ArcGIS 9.3) and proximity between these points and the wildebeest polygons was also measured in the same way as the actual points [44]. Because migratory wildebeest herds represent by far the greatest biomass of the migratory ungulate species, we have chosen to focus our analysis on this species. In addition, the movements of other migratory species, such as Burchell’s zebra and Thomson’s gazelle herds, and accompanying carrion from these sources, follow similar patterns to the migratory wildebeest herds [45].

Normalized Difference Vegetation Index (NDVI) is a reliable measure of greenness or wetness and is linked to rainfall and forage availability, and thus mortality, for many ungulate species [18], [41]. To assess the relationship between vulture locations and prey mortality, we related vulture movements to a Normalized Difference Vegetation Index (NDVI). NDVI values were extracted for centroids of the day range of vulture points and a random set of points from within the minimum convex polygon of each individual’s range in ArcGIS 9.3. Information on vegetation indices from MOD13Q1 were obtained from http://lpdaac.usgs.gov/get_data maintained by the NASA Land Processes Distributed Archive Center (LP DAAC), USGS/Earth Resources Observation and Science (EROS) Center, Sioux Falls, South Dakota in January 2012. These data provide 16-day composites of vegetation indices at 250-meter spatial resolution. High NDVI values can be indicative of either high tree cover or high grass cover. Thus, data points were also related to tree cover using data from Guan et al. [46], and all points with greater than 60% tree cover were excluded from analysis.

### Statistical analysis

We used a linear mixed-effects model to assess patterns of vulture habitat use. To determine habitat selectivity in relation to wildebeest, values were averaged across month to reduce issues of pseudo-replication. Two models were run – one for wildebeest and one for NDVI. For both models, the dependent variable was calculated as the real values minus the randomly generated values of either proximity to wildebeest or NDVI. Therefore, values near zero suggest the distribution of vulture movement is no different from random, while negative values suggest vultures are close to wildebeest (or in relatively dry areas) and positive values suggest vultures are far from wildebeest (or in relatively wet areas). Models included season (dry, dry to wet, wet, or wet to dry), species (Ruppell’s, White-backed, or Lappet-faced vulture), and breeding status of the individual (used nest or did not use nest) as fixed factors with unit id as a random factor to account for differences between individuals using lme4 package [47]. AIC values were used to select the best model in a forward stepwise method. All statistical analyses were preformed in R 2.7.2 (R Development Core Team 2008). Means and standard error are provided. Analyses of habitat preference follow Johnson’s [48] third order of selection, where habitat availability is determined based on home range size.

## Results

Data included in the analysis came from 39 vultures tracked for an average of 149 days from which sufficient data were collected (Table 1). Twenty-one of the birds studied had active nests, and 962 days of “observation” were excluded due to birds being on the nest for at least one of the three mid-day points. On average, the centroids of daytime vulture locations overlapped with wildebeest migratory herds 31% (±0.01%) of the time. The proportion of days during which vultures overlapped with wildebeest was highest during the dry season (60%±0.01%).

**Table 1:** Sample size by species.

Species	Individuals	# of Juveniles	Days	Average days per individual
Ruppell's vulture	15	1	1800	120
White-backed vulture	12	4	2276	190
Lappet-faced vulture	12	6	1747	146

**Figure 1:**
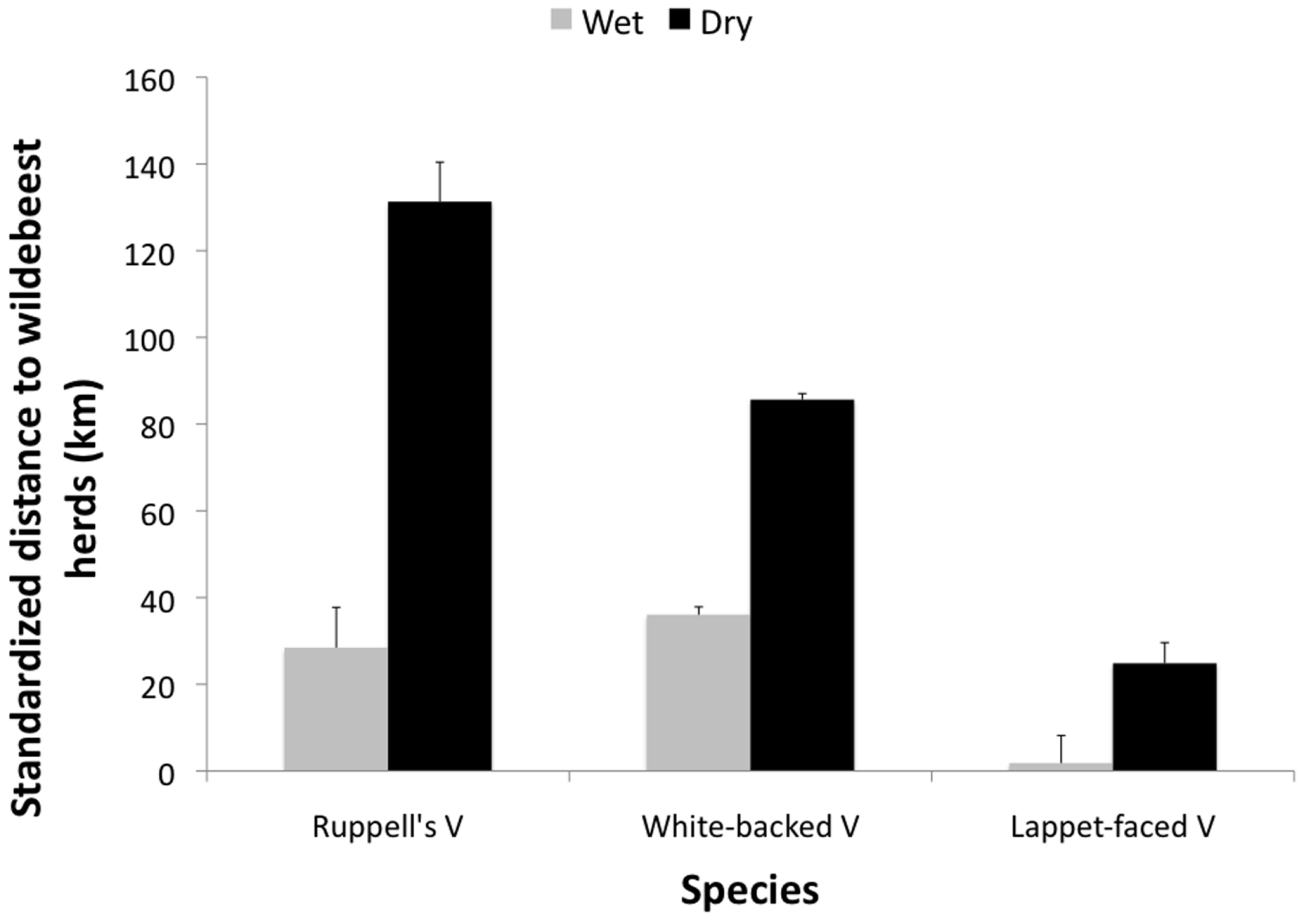
Random point distance minus vulture data point distance to wildebeest herds (km) by season and species. Note: Values near zero suggest the distribution of vulture movement is no different than random, while positive values suggest vultures are closer to wildebeest than by chance alone and negative values would suggest vultures are farther from wildebeest than by chance alone.

**Figure 2:**
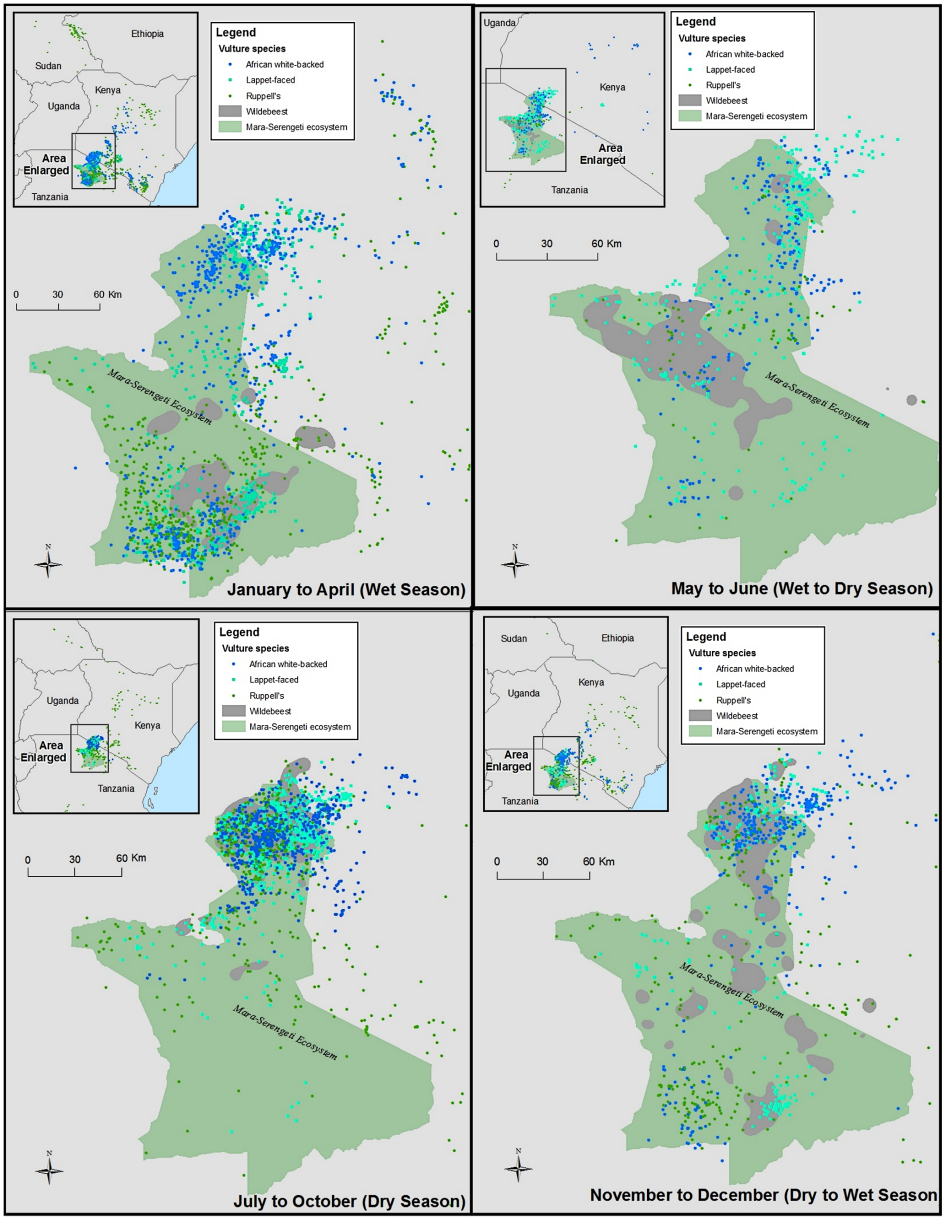
Vulture movement in relation to wildebeest migration across four seasons.

All three species showed a significant preference for being closer to wildebeest herds only in the dry season (Figure 1). In addition, all three species used areas where migratory ungulates never occurred and the two *Gyps* species, in particular, frequented a number of areas beyond the Mara-Serengeti ecosystem including both Tsavo National Parks in Kenya and Northern Kenya (Figure 2). One Ruppell’s vulture spent three months in the Boma-Jonglei area in Sudan-Ethiopia, where a separate migratory ungulate population of white-eared kob (*Kobus kob*) occurs [49].

**Figure 3:**
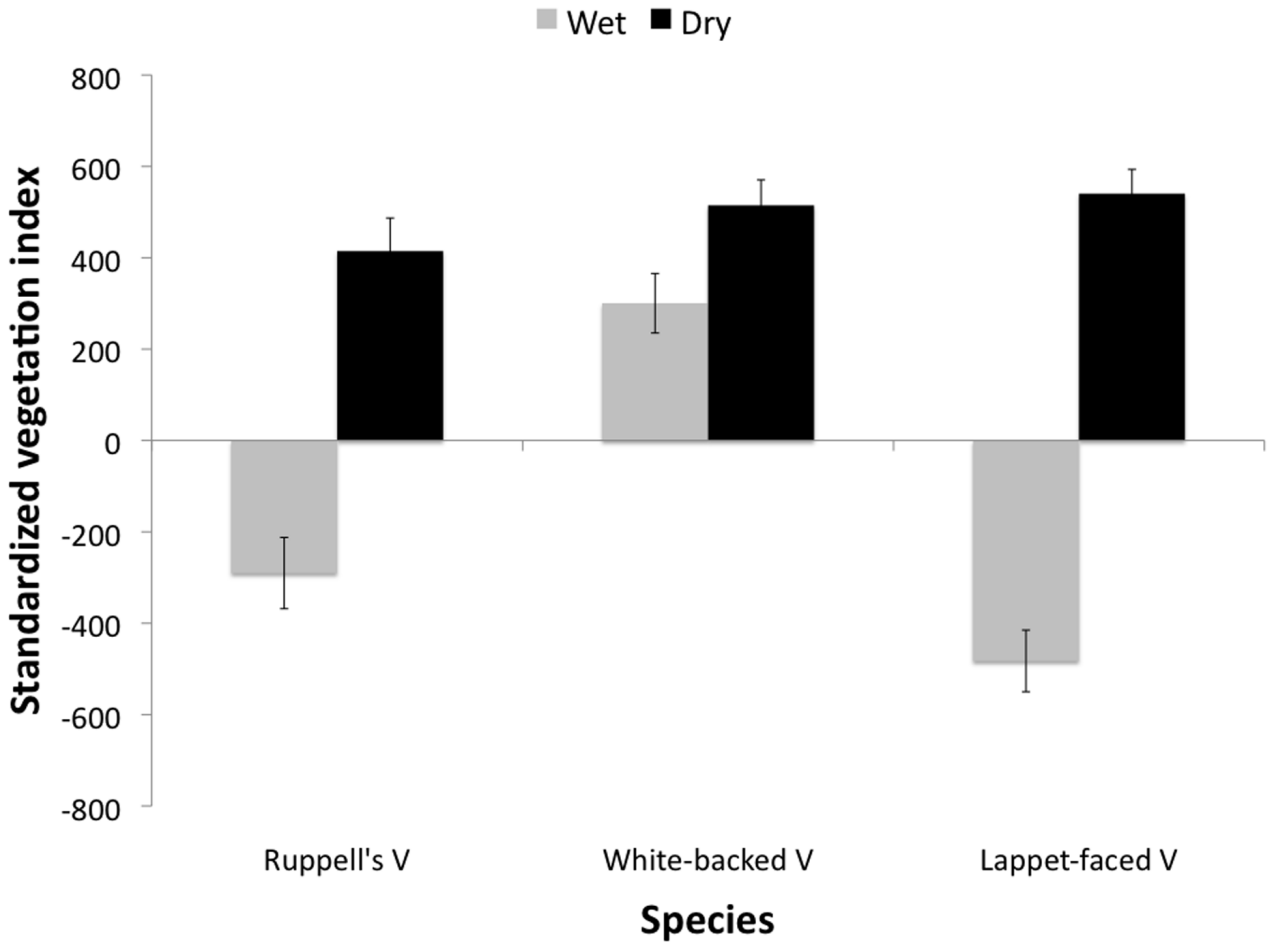
Standardized vegetation index (based on NDVI values of real vulture points minus NDVI values of background points) by species and season. Note: Values near zero suggest the distribution of vulture movement is no different than random, while negative values suggest vultures are in relatively dry areas and positive values suggest vultures are in relatively wet areas.

Vultures being near migratory wildebeest herds, in relation to NDVI, was significantly affected by season, species, and breeding status (Table 2). White-backed vultures showed the greatest selectivity for wildebeest, followed by Ruppell’s and Lappet-faced vultures, respectively. All three species preferred greener areas during the dry season, and White-backed vultures preferred greener areas in the wet and dry to wet seasons (Figure 3). Ruppell’s vultures and Lappet-faced vultures selected browner areas in the wet season. Breeding vultures tended to be farther from the herds and in drier areas than were non-breeding individuals.

**Table 2:** GLMM model for habitat selectivity in relation to proximity to wildebeest and NDVI.

Variables	Wildebeest (AIC = 74462)	NDVI (AIC = 101156)
Intercept	–16.5 (21.3)	210.7 (149.45
Season (Wet to dry)	–17.2 (7.0)	195.6 (82.8)
Season (Dry)	–78.4 (4.9)	661.6 (58.9)
Season (Dry to wet)	–13.4 (5.2)	219.6 (62.3)
Species (Ruppell’s)	10.8 (24.4)	–476.7 (170.9)
Species (Lappet-faced)	41.4 (25.8)	–325.5 (177.9)
Breeding Status (breeding)	7.4 (21.1)	–333.0 (147.3)
Number of individuals	39	38
Number of days	5823	5708

Note: Parameter estimate (and standard error) given for all variables included in the model. Base values (for dummy variables) are for non-breeding White-backed vultures in the wet season.

## Discussion

### Vulture habitat use is not driven by prey abundance

Carcass availability is mediated by both prey abundance and prey mortality. In our study area, vulture habitat use is not driven by abundance of live ungulates. Despite the fact that migratory wildebeest herds consistently represent the greatest prey abundance in this landscape, vultures selectively associate with them only during the dry season. Vultures showed limited preference for being near migratory herds, with overlap between vultures and wildebeest migratory herds being limited to less than 30%, contrary to previous studies [15], [21].

Our study suggests that prey mortality may be a more important driver of vulture habitat use than prey abundance. As predicted, vultures showed greater use of migratory herds during the dry season, when migratory-herd mortality is high [20]. Abundance of migratory herds is stable throughout the year, but there are dramatic shifts in mortality depending on season. The fact that the dry season was the only period when vultures showed selectivity to be near migratory herds strongly suggests vulture habitat use, and the preference for being close to herds, is affected by mortality, and subsequently carcass availability rather than by the abundance of live ungulates. In addition, two species of vultures, Lappet-faced and Ruppell’s, preferentially selected dry or brown areas during the wet season. Rainfall and forage availability are known to have major impacts on ungulate survival [41]. Drier areas should lead to higher mortality in ungulates, although this connection merits further study. The selection of dry areas during the wet season and lack of selection of migratory herds is thus consistent with prey mortality being more important than prey abundance in driving scavenger habitat use.

Seasonal shifts in ranging behavior, particularly wider ranging of White-backed vultures during the wet than dry season, is consistent with movement studies in Southern Africa [36]. The vultures’ breeding season partially overlaps with the period of heavy use of migratory herds. While vultures may alter foraging behavior while breeding, generally reducing foraging frequency (i.e. the number of days on which they forage), they have not been found to reduce the overall distance travelled or area covered during the breeding season [50], [35]. Because of their energy efficient soaring flight, vultures are capable of following wildebeest herds at very low energetic costs [13], [51]. Additional data from this study and other movement research on vultures indicate that vultures can often travel greater than 100 km in a day, making it possible for even cliff-nesting species to access herds throughout the year [36], [35]. Use of migratory herds, which provide a consistent, more aggregated food source during the dry season, may be particularly important to breeding vultures that are limiting foraging effort. That said, high levels of individual differences in habitat use make vulture movement behavior inconsistent with central place foraging theory [52]. Thus it is unlikely that vultures use migratory herds during the dry season solely because of limitations to movement that might accompany breeding. And indeed, Ruppell’s vultures actually have to travel farther to reach wildebeest herds during the breeding season, which typically overlaps with the dry season, as there are no cliff sites near Masai Mara National Reserve [30], [28].

### Differences in habitat use among the three species may enable coexistence

Differences in habitat use among these three species may enable coexistence. Lappet-faced vultures showed less selection for migratory wildebeest than *Gyps* vultures, likely due to differences in wing-loading and use of small as well as large carrion sources [15], [21], [53]. Ruppell’s vultures showed slightly but not significantly lower use of migratory herds than did White-backed vultures, which may be due to the fact that Ruppell’s nest in cliffs which are often hundreds of kilometers from the wildebeest migration and, may thus use other foraging areas of similar or lower quality in closer proximity to their nests.

White-backed vultures selected relatively green areas during the wet season, unlike Lappet-faced and Ruppell’s vultures, suggesting that different factors may drive wet-season habitat use in this species. Reasons for this remain unclear. White-backed vultures may use slightly different foraging strategies than the other two species, perhaps with closer dependence on prey abundance, as evidenced by their higher selectivity to be near migratory herds. This difference in large-scale habitat use may be critical for the coexistence in the two *Gyps* species, particularly during periods of reduced food availability as occurs during the wet season.

### Conservation implications

Poisoning of carrion resources, typically done by pastoralists to kill predators, is believed to be the primary threat to vultures at our study site (Kendall and Virani, 2012). Nevertheless, declines in food availability have led to rapid declines in vulture populations elsewhere [54]. In East Africa, vultures are likely dependent on the persistence of both migratory herds, during the dry season, and resident ungulates, during the wet season. Vultures generally fledge chicks during the dry season [27], [28]. Given that fledgling success is highly dependent on food availability, declines in migratory herds may impact vulture populations [29], [53], with threats to migratory herds and changes in their dry-season range affecting the survival of these birds [55], [39]. In addition, food availability may be an important limiting factor during the wet season, when ungulate mortality rates are low [20]. Whereas the effect of important human-mediated habitat factors, such as the management of protected areas, human settlement densities, and the numbers and locations of powerlines, on vulture movements was not the focus of our study, on-going research suggests that vultures preferentially use protected areas throughout the year (Kendall, unpublished data). Combined with the fact that current livestock management practices in Kenya limit the availability of livestock carrion to scavengers, vultures appear to depend upon resident wildlife populations for carrion during the non-dry season (Reson & Kendall, unpublished data). Given that resident wildlife populations are declining rapidly throughout Kenya [56], particularly in Masai Mara National Reserve [55], [57], food availability is likely to become a major issue for vulture survival in the near future as has occurred elsewhere [54].

Concentrations of vultures around migratory herds during the dry season may offer a significant opportunity for monitoring vulture populations. Because all vultures used in this study frequented the wildebeest herds throughout the dry season, roadside counts done in this area during this period may give the most accurate and cost-effective account of the population status of *Gyps* vultures in East Africa. The rapid declines in these species that are now underway, coupled with difficulties in assessing population status of wide-ranging vertebrates, suggest that such monitoring should continue [32].
